# Chronic mucocutaneous candidiasis associated with paracoccidioidomycosis in a patient with mannose receptor deficiency: First case reported in the literature

**DOI:** 10.1590/0037-8682-0008-2021

**Published:** 2021-03-22

**Authors:** Dewton de Moraes Vasconcelos, Dalton Luís Bertolini, Maurício Domingues Ferreira

**Affiliations:** 1 Universidade de São Paulo, Faculdade de Medicina, Hospital das Clinicas, Departamento de Dermatologia, Ambulatório das Manifestações Cutâneas das Imunodeficiências Primárias, São Paulo, SP, Brasil.

**Keywords:** Chronic mucocutaneous candidiasis, Paracoccidioidomycosis, Mannose receptor deficiency

## Abstract

We describe the first report of a patient with chronic mucocutaneous candidiasis associated with disseminated and recurrent paracoccidioidomycosis. The investigation demonstrated that the patient had a mannose receptor deficiency, which would explain the patient’s susceptibility to chronic infection by *Candida* spp. and systemic infection by paracoccidioidomycosis. Mannose receptors are responsible for an important link between macrophages and fungal cells during phagocytosis. Deficiency of this receptor could explain the susceptibility to both fungal species, suggesting the impediment of the phagocytosis of these fungi in our patient.

## INTRODUCTION

Chronic mucocutaneous candidiasis (CMC) is a rare primary immunodeficiency characterized by persistent and refractory infections of the skin, attachments, and mucous membranes caused by *Candida* spp. These fungal infections are usually restricted to the mucocutaneous surface, although in very rare cases, they are associated with systemic disease or septicemia. Different mechanisms have been described that address defects in cytokine production and the response of monocytic efferent cells to *Candida* spp^1^. Paracoccidioidomycosis is the main endemic type of systemic fungal infection in Brazil, and its evolution and clinical conditions are closely linked to the host’s immunological status. We report the case of a patient with CMC associated with paracoccidioidomycosis, with an unusual evolution, probably related to the patient’s peculiar immunological status. We conducted a search for English-language articles in the following databases: PubMed, Google Scholar, Scielo, Current Contents/Clinical Medicine, and Scopus, with the following keyword associations: “chronic mucocutaneous candidiasis and mannose receptor deficiency,” “chronic mucocutaneous candidiasis and paracoccidioidomycosis,” “paracoccidioidomycosis and mannose receptor deficiency,” and “chronic mucocutaneous candidiasis and paracoccidioidomycosis and mannose receptor deficiency.” However, no case reports were found. Therefore, we conclude that our case is the first case report in the medical literature, which reveals a previously undescribed new form of clinical susceptibility to paracocciodomycosis as well as a new pathophysiological mechanism of susceptibility to chronic mucocutaneous candidiasis and paracoccidioidomycosis.

## CASE REPORT

A 43-year-old woman from the interior of the state of São Paulo, Brazil, who showed normal healthy neuropsychomotor development up to 15 years of age, began presenting oral candidiasis, esophageal candidiasis, persistent nail mycoses, and recurrent vaginal candidiasis. CMC was diagnosed and showed poor response to oral antifungals. At age 30, the patient developed fever, weight loss, and cervical polyadenopathy for a few weeks. A lymph node biopsy was performed, which showed a granulomatous inflammatory process with areas of necrosis and giant cells as well as fungal images of *Paracoccidoides brasiliensis*. Ganglionic paracoccidioidomycosis was diagnosed. She was treated with amphotericin and itraconazole and was soon asymptomatic. Five years later, she presented with daily fever, weight loss, left cervical and axillary polyadenopathy, and a cutaneous lesion on her left shoulder that was associated with significant pain when moving her left arm. She underwent ganglion and skin biopsies again, and an additional joint puncture was performed. *Paracoccidioides brasiliensis* was isolated from all three biopsies, indicating cutaneous, joint, and ganglionic paracoccidioidomycosis. Itraconazole was prescribed for 18 months, resulting in complete remission of the signs and symptoms. CMC was completely resolved during treatment with itraconazole. Seven months after completing itraconazole treatment, she was able to adequately control oral candidiasis solely with oral nystatin. However, she again presented with fever, weight loss, and suspected reactivation of paracoccidioidomycosis. Itraconazole was again prescribed, with complete remission of symptoms. Six months later, even during itraconazole treatment, symptoms of fever, weight loss, hepatomegaly, and polyadenopathy reappeared. She then underwent a liver biopsy, and a visceral form of paracoccidioidomycosis was diagnosed. She was treated with amphotericin, which was replaced with 800 mg sulfamethoxazole and 160 mg trimethoprim twice daily. She has been prescribed these drugs for five years and has shown no recurrence of paracoccidioidomycosis. Oral and vaginal candidiasis has been adequately controlled by oral and vaginal nystatin. There is no prospect of withdrawing antifungals. Her family has several cases of consanguineous unions: her paternal parents and grandparents were second cousins, and her paternal great-grandparents were first cousins. She had nine siblings, including two sisters who presented with recurrent oral candidiasis and a brother with chronic nail fungus. 

### Examinations

The patient presented negative serology for HIV 1/2, hepatitis C, and hepatitis B. Levels of glucose, urea, creatinine, uric acid, protein electrophoresis, immunoglobulin, CH50, complement C3 and C4, ferritin, blood count, and platelets were all normal. She did not present with paraproteinemia. A monoclonal antibody assay and evaluation by flow cytometry showed that the expression of interleukin 12 (IL-12) and interferon-gamma (IFN-γ) receptors in lymphocytes and monocytes was normal and compatible with a control group of healthy individuals. Lymphocyte phenotyping showed normal indices (Table 1).

**TABLE 1:** Lymphocyte phenotyping indices.

Lymphocyte phenotyping Cells	Relative (%)	Absolute (/mm^3^)
Total Leucocytes		8000
Total Lymphocytes	29.20	2392
Lymphocytes T CD3+	81.70	1954
Lymphocytes T CD4+	52.80	1263
Lymphocytes T CD8+	28.90	691
Lymphocytes CD19+	8.20	196
CD4+CD45RA+	22.90	548
CD4+CD45RO+	26.50	634
CD3+CD56+	10.50	251
CD3-CD56+	7.20	172
CD4+/CD8+	1.83	

Evaluation of the proliferative capacity of T lymphocytes showed a normal stimulation index when stimulated by phytohemagglutinin, pokeweed mitogen, anti-CD3 and cytomegalovirus antigens, and *Candida* metabolic antigen (CMA).

Our patient participated in a study (unpublished) in which intracellular cytokine production from lymphomonocytic cells after stimulation with tetanus toxin (TT) or *Candida* antigens was investigated in 15 patients with CMC and 13 healthy controls. The production of cytokines (IL-2, IL-4, IL-10, and IFN-γ) of total T cells was evaluated using a monoclonal antibody assay and flow cytometry. Results indicated that patients with CMC could be divided into two subgroups after stimulation by *Candida*, tetanus, or unstimulated antigens. The first subgroup showed a significant decrease in T cells producing IL-2, IFN-γ, IL-4, and IL-10 compared to the healthy control group. The second subgroup showed a significantly higher number of IL-2 and IFN-γ-producing T cells than observed in the first subgroup, similar to the control group. Our patient was placed in the latter subgroup with a significantly increased number of IL-2 and IFN-γ-producing T cells. 

**FIGURE 1:**
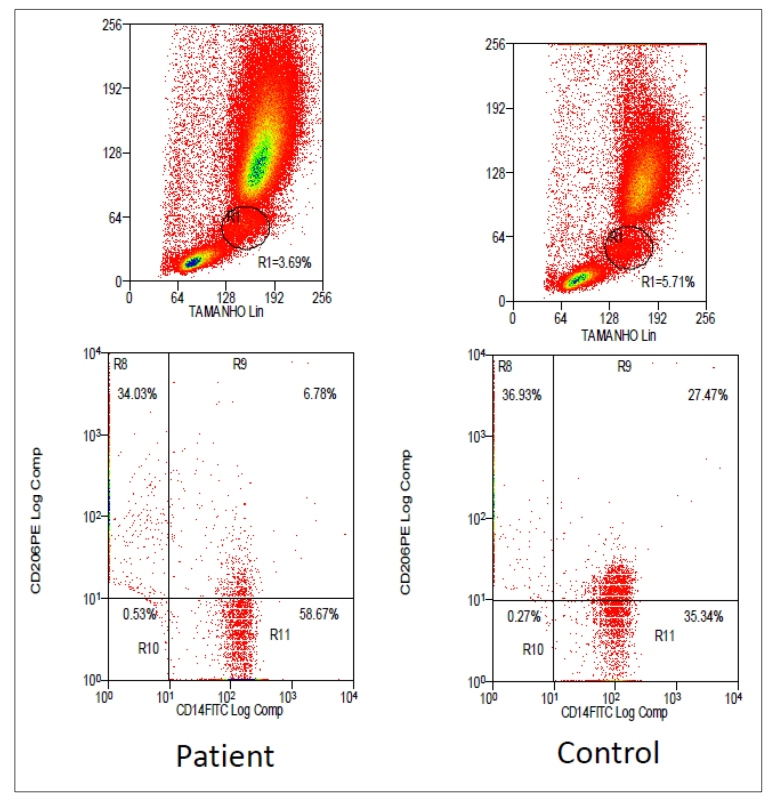
Graphs showing CD206 expression in the monocytes of the patient and healthy control. A significant decrease in CD206 expression was observed in the patient's monocytes compared with those of the healthy control.

 No deficiency in TH1-type cytokine production (IL-2 and IFN-γ) from monolymphocytic cells was verified after stimulation with CMA, tetanus toxoid antigen, and unstimulated antigen in this patient. Additionally, we evaluated the number of mannose receptors in monocytes using an assay with a monoclonal antibody specific for CD206 (mannose receptor) and flow cytometry, which revealed a significant decrease in the patient’s mannose receptors compared to the healthy control. Our patient presented with 6.78% of CD206 expression in monocytes and 27.47% in healthy control monocytes. (Figure 1).

## DISCUSSION

Our patient’s susceptibility to mucocutaneous candidiasis and its chronicity is characteristic of CMC^1^ ^,^ ^2^. She was also susceptible to *P. brasiliensis*. The association between CMC and invasive fungal infections has been very rarely reported in the literature. CMC almost always remains limited to the skin, attachments, and mucous membranes. There are few reports of invasive fungal infections in patients with CMC. Meningitis due to *Candida albicans*, *Cryptococcus neoformans*, and histoplasmosis are among the exceptions reported^3^ ^,^ ^4^. No cases of CMC associated with paracoccidiomycosis have been described. *P. brasiliensis* is characterized by a typical latency period and reappearance during an immunodeficient state^5^. All episodes of paracoccidioidomycosis presented by the patient were invasive and disseminated, affecting the lymph nodes, joints, and liver. These are important features of an immunodeficiency. Absence of anti-fungal defense was probably caused by a genetic defect. Genetic inheritance is evident in this case, since the patient is a product of three generations of consanguineous unions and has three siblings with some degree of susceptibility to mucocutaneous candidiasis.

Paracoccidioidomycosis is a granulomatous disease, and macrophages are the main cellular defense against the pathogen^6^. The literature describes variable susceptibility to paracoccidioidomycosis development in mice and humans. This is likely genetically determined^7^ ^,^ ^8^. This susceptibility suggests an imbalance in the secretion of cytokines that inhibit the host’s cellular immune response^8^ and macrophage function^7^. Some studies have reported that resistance to *P. brasiliensis* in mice is related to the immune response T-helper 1 (Th1) with high secretion of IL-2 and IFN-γ^8^ ^,^ ^9^, and susceptibility is associated with very low levels of these cytokines^8^ ^,^ ^10^. This mechanism has also been identified in mice with resistance or susceptibility to candidiasis and in patients with CMC^2^.

Although our patient was unable to conquer *Candida* infections in the mucous membranes and showed susceptibility to invasive infections by *P. brasiliensis*, we observed no deficiencies in IL-2 or IFN-γ production after stimulation with either CMA or TT. Similarly, no changes were observed in the expression of IL-12 and IFN-γ receptors. Such changes would have elegantly explained our patient’s susceptibility to fungi.

In contrast, we observed a significant decrease in mannose receptors in the patient’s monocytes compared to that in healthy control. The mannose receptor (CD206) is a type C lectin found on the surface of immature macrophages and dendritic cells, human dermal fibroblasts, and keratinocytes^11^. Mannoses are an important constituent of the fungal wall. Mannoproteins are the main constituents of the outer layer of yeast cell walls and are important in the opsonic-independent interaction with phagocytic cells through mannose receptors and for inducing yeast intake by professional phagocytes. Mannose receptors also mediate endocytosis of soluble ligands by means of clathrin-coated pits^12^. This raises an interesting hypothesis: our patient probably presents deficient resistance to infection by *Candida* and *P. brasiliensis* due to the low expression of mannose receptors in monocytic cells, which causes a specific defect in the phagocytosis of these two fungi. Lymphoproliferation tests indicated that the patient was able to activate lymphocytes, including in response to CMA; produce Th1-type cytokines (IL-12 and IFN-γ); and mobilize macrophages, since biopsies demonstrated the formation of granulomas. It seems very likely that her macrophages have an insufficient interaction with the fungi because of deficient numbers of mannose receptors, making phagocytosis unfeasible and probably resulting in susceptibility to *Candida* spp. and *P. brasiliensis*. 
